# Development of a Portable All-Wavelength PPG Sensing Device for Robust Adaptive-Depth Measurement: A Spectrometer Approach with a Hydrostatic Measurement Example

**DOI:** 10.3390/s20226556

**Published:** 2020-11-17

**Authors:** Shao-Hao Chen, Yung-Chi Chuang, Cheng-Chun Chang

**Affiliations:** Department of Electrical Engineering, National Taipei University of Technology, Taipei 10608, Taiwan; t106318147@ntut.edu.tw (S.-H.C.); luca790305@gmail.com (Y.-C.C.)

**Keywords:** all-wavelengths, PPG, hydrostatic pressure

## Abstract

Photoplethysmography (PPG), a noninvasive optical sensing technology, has been widely used to measure various physiological indices. Over-the-counter PPG devices are typically composed of a single-wavelength light source, namely, single-wavelength PPG (SW-PPG). It is known that signals of SW-PPG are easily contaminated or distorted by measurement conditions such as motion artifacts, wearing pressure, and skin type. Since lights of different wavelengths can penetrate skin tissues at different depths, how to effectively construct a multiwavelength PPG (MW-PPG) device or even an all-wavelength PPG (AW-PPG) device has attracted great attention. There is also a very interesting question, that is, what could be the potential benefits of using MW-PPG or AW-PPG devices? This paper demonstrates the construction of an AW-PPG portable device and conducts a preliminary evaluation. The presented device consists of four light-emitting diodes, a chip-scale spectrometer, a microcontroller, a Bluetooth Low Energy transceiver, and a phone app. The maximum ratio combining algorithm (MRC) is used to combine the PPG signals derived from different wavelengths to achieve a better signal-to-noise ratio (*S*/*N*). The PPG signals from the developed MRC-AW-PPG device versus those from the conventional SW-PPG device are compared in terms of different hydrostatic pressure conditions. It has been observed that the MRC-AW-PPG device can provide more stable PPG signals than that of a conventional PPG device. The results shine a light on the potential benefits of using multiple wavelengths for the next generation of noninvasive PPG sensing.

## 1. Introduction

Photoplethysmography (PPG) is a noninvasive optical sensing technology that records vascular volume change. PPG can help people to recognize their health conditions, such as heart rate (HR), heart rate variability (HRV), hemoglobin oxygen saturation (SpO2), and atrial fibrillation (AF). Due to wide applications, most wearable devices have been embedded with PPG sensors. In Q3 2019, the worldwide shipments of wearable devices achieved 84.5 million units, a year-over-year increase of 94.6% and a new record for shipments in a single quarter [1]. In 2020, the worldwide end-user spending on wearable devices is expected to reach $52 billion [2]. As the wearable devices market grows, PPG devices will become more common. However, over-the-counter PPG devices are typically composed of a single-wavelength light source, namely, single-wavelength PPG (SW-PPG). It is known that signals of SW-PPG are easily contaminated or distorted by measurement conditions such as motion artifacts, wearing pressure, and skin type.

The pressure on the blood vessel is one of the more interesting issues. There are three types of pressure on the blood vessel, namely, hemodynamic pressure, contact pressure, and external hydrostatic pressure. The hemodynamic pressure comes from the heartbeat. The contact pressure is related to the force of the sensor touching the skin. In [3,4,5,6], it has been noted that the most suitable wavelength changes with the contact pressure. The contact pressure, in most scenarios, can be considered a constant, since the user will not intentionally press the device when the device is worn. The hydrostatic pressure is a force generated by the pressure of the fluid on the capillary walls, which leads to major changes in the PPG signal. In the literature [7], it has been noted that the sharp peak of PPG waveforms under negative hydrostatic pressure is significantly rounder than the similar peak under positive hydrostatic pressure. In [8,9,10,11,12], it has been noted that the hydrostatic pressure tends to cause errors in the blood pressure assessment. The hydrostatic pressure is likely to cause changes when people perform activities and swing their hands. The sum of the above three pressures will cause perturbation in the pulsatile signals. Hence, the most suitable depth for measuring PPG signals needs to be adaptively changed.

**Figure 1:**
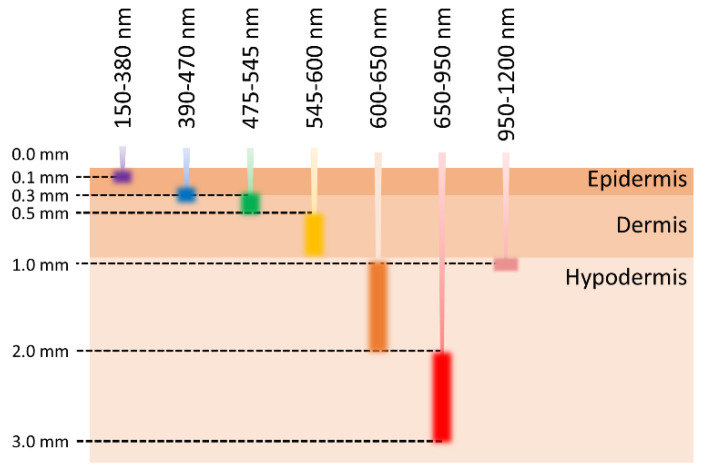
The relationship between light wavelengths and penetration depths.

Single-wavelength, and consequently measuring at a constant depth, is one of the main aspects of PPG that limits the accuracy of SW-PPG devices when it comes to recording PPG signals in daily life. In the literature [13,14,15], it has been noted that different wavelengths can penetrate skin tissues at different depths, as shown in Figure 1. How one might effectively construct a multiwavelength PPG (MW-PPG) device, and even an all-wavelength PPG (AW-PPG) device, has attracted great attention. There is also a very interesting question, that is, what are the potential benefits of using MW-PPG or AW-PPG equipment?

**Figure 2:**
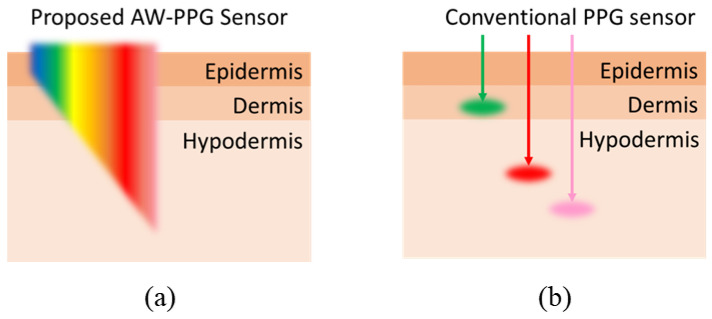
Illustration of sensing ability of all-wavelength photoplethysmography (AW-PPG) sensors against conventional PPG sensors: (**a**) AW-PPG sensors; (**b**) Conventional PPG sensors.

This paper demonstrates a construction of AW-PPG portable devices and conducts a preliminary evaluation. AW-PPG devices are expected to record the pulsatile signal at adaptive depths and hence are expected to improve the PPG signals in different hydrostatic pressure conditions, as illustrated in Figure 2. At present, there is no available over-the-counter PPG sensing device that can support AW-PPG measurement. Current PPG sensing devices may use a broad-spectrum photodetector to record different PPG wavelength signals in a sequential sampling manner [16,17,18,19]. However, the number of sensing channels is very limited.

In this study, we integrated a chip-scale spectrometer, a microcontroller (MCU), and a Bluetooth Low Energy (BLE) transceiver to develop a portable AW-PPG measurement device. The dimension of the developed proof of concept AW-PPG device is 38 mm × 25 mm × 15 mm. It is compact and lightweight. This device can record a large number of PPG signals at different wavelengths with one broad-spectrum light-emitting diode (LED) or a few LEDs covering broad-spectrum. Furthermore, we embedded the maximum ratio combining algorithm into the MCU. The algorithm combined different wavelength signals, that is, signals from different depths, to achieve a better signal-to-noise ratio *(*S/N). A mobile app was implemented to acquire the PPG signal in real time. Finally, we examined the change of *S/N* (∆S/N) with the newly developed AW-PPG device against conventional PPG devices. The potential benefit of AW-PPG was observed.

The organization of the rest of this paper is stated as follows. Section 2 introduces the design of the AW-PPG device. Section 3 explains the signal processing algorithm. Section 4 shows the result of comparing the developed novel AW-PPG sensor against the conventional PPG device in different scenarios and discusses the potentials of the AW-PPG device, and we conclude in Section 5.

## 2. AW-PPG Device

**Figure 3:**
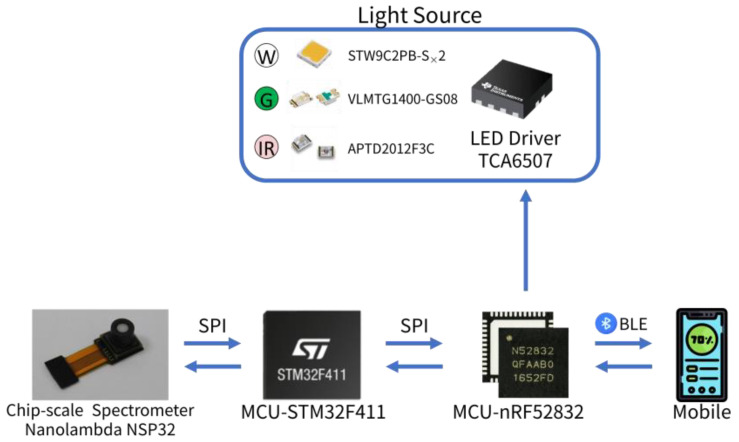
The proposed AW-PPG architecture.

The focus of this study was to develop a real-time synchronic all-wavelength PPG device. The architecture of the proposed AW-PPG device is shown in Figure 3.

**Figure 4:**
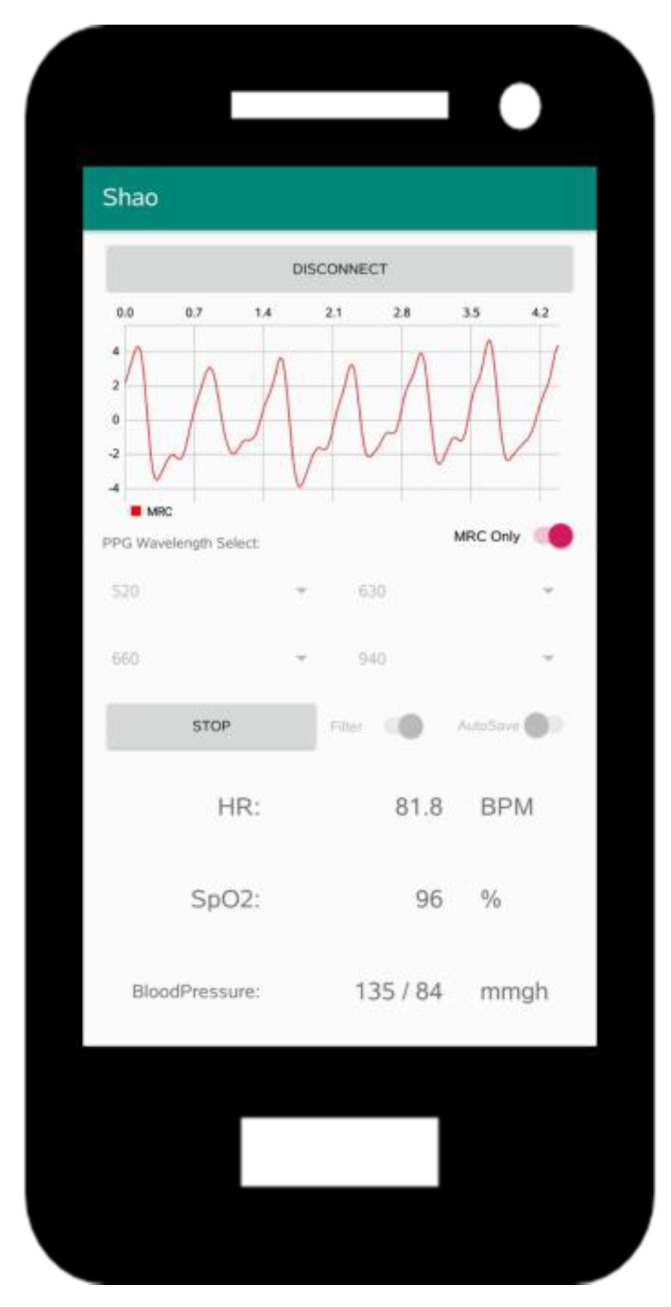
The developed mobile app.

We designed a mobile app to communicate with the proposed AW-PPG device via Bluetooth Low Energy (BLE) and acquire the PPG signals in real time. As illustrated in Figure 4, we used a mobile device to assess the physiological information, such as heart rates, SpO2, and blood pressure, using algorithms developed in our previous work [20].

**Figure 5:**
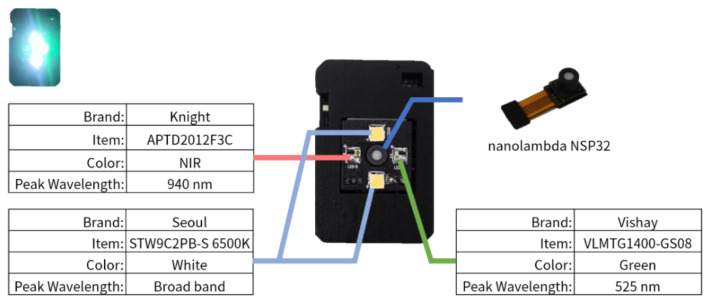
Light source specs and configuration.

**Figure 6:**
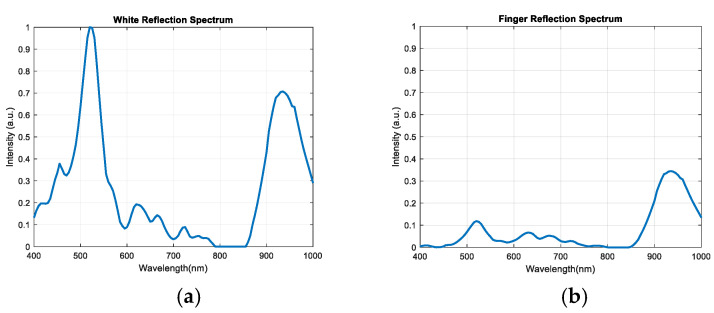
Reflection spectrum: (**a**) on a standard reflection white plate; (**b**)on a finger.

The nRF52832 (Nordic Semiconductor) is responsible for transferring command from a mobile device to the MCU, STM32F411 (STMicroelectronics), and returning PPG signals to the mobile device. The nRF52832 also controls four LED light sources. As shown in Figure 5, we used two white LEDs, a green LED, and a Near-infrared (NIR) LED. The spectrum of the light sources is shown in Figure 6a. The white LED covered the wavelengths in the visual range, the green LED enhanced the spectrum intensity at the wavelengths where the used white LED largely decayed, and the NIR LED was used to try to cover the wavelengths in the NIR range. The reflection spectrum of the designed device is shown in Figure 6, on both (a) a standard reflection white plate, and (b) a finger.

**Figure 7:**
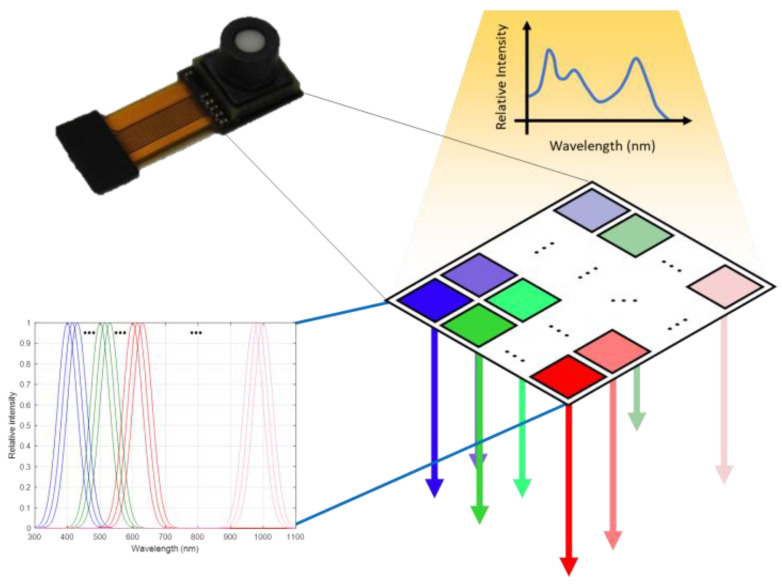
Structure of the used chip-scale spectrometer.

STM32F411 is responsible for controlling the spectrometer sensor and for sending the PPG data from the sensor to nRF52832. We selected the chip-scale spectrometers, NSP32 (nanolambda, Daejeon, Korea), from nanoLambda, as the AW-PPG sensor. The core technology of this sensor is based on plasmonic filters, which can be integrated into a regular photodetector such as a complementary metal-oxide semiconductor (CMOS) imager. For more details, please refer to our previous work 20. Briefly, it can be considered that the sensor is composed of many narrow-spectrum photodetectors. The advantage of this sensor is its small chip-scale size and its ability to sense the spectrum in a wide range of 400 to 1000 nm. We used NSP32 with 4 LEDs as our light source, covering broad-spectrum, to implement a portable adaptive-depth AW-PPG device, as illustrated in Figure 7.

## 3. Algorithm: Maximum Ratio Combining and Filtering

The maximum ratio combining (MRC) is an optimum diversity combining technique. It gives the weighting to the signals from each channel and combines them together. We could thus view the spectrum of each wavelength as the signals from each channel. The spectrum of each wavelength can be defined as

(1) $$y_{\lambda_{i}}=s_{\lambda_{i}}+n_{\lambda_{i}}$$

where $\lambda_{i}\;$ means 400 nm, 405 nm, …, 995 nm and 1000 nm, i = 1, …, 121, $s_{\lambda_{i}}$ is the signal component, $n_{\lambda_{i}}$ is the white noise component. The *S/N* at $\lambda_{i}$ can be defined as

(2) $$S/N_{\lambda_{i}}=\frac{E\left[s_{\lambda_{i}}{}^{2}\right]}{E\left[n_{\lambda_{i}}{}^{2}\right]}=\frac{\mu_{\lambda_{i}}{}^{2}}{\sigma_{\lambda_{i}}{}^{2}}$$

**Figure 8:**
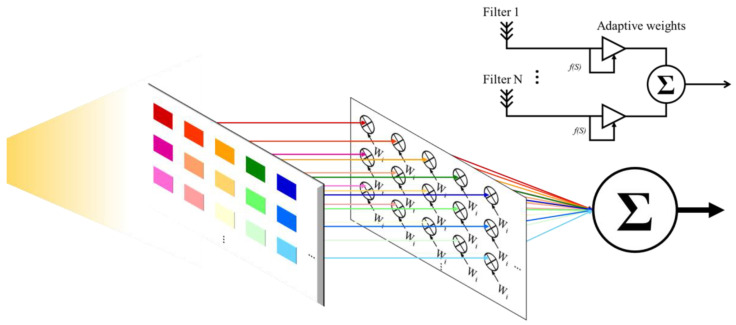
Maximum ratio combining (MRC) architecture in PPG measurement.

We proposed to combine all wavelengths with an MRC approach, namely, MRC-AW-PPG signal, as shown in Figure 8.

The weighting $w_{i}$ and the full-wavelength combined signal $y_{MRC}$ can be associated as

(3) $$y_{MRC}=\overset{121}{\underset{i=1}{\sum }}w_{i}y_{\lambda_{i}}=\overset{121}{\underset{i=1}{\sum }}w_{i}s_{\lambda_{i}}+\overset{121}{\underset{i=1}{\sum }}w_{i}n_{\lambda_{i}}=s_{MRC}+n_{MRC}$$

Assuming noise terms are uncorrelated, the signal power and noise power of the combining signal can be expressed as

(4) $$\{\begin{array}{c} E\left[s_{MRC}{}^{2}\right]=E\left[{\left(\overset{121}{\underset{i=1}{\sum }}w_{i}s_{\lambda_{i}}\right)}^{2}\right]\\ E\left[n_{MRC}{}^{2}\right]=E\left[{\left(\overset{121}{\underset{i=1}{\sum }}w_{i}n_{\lambda_{i}}\right)}^{2}\right]=E\left[\overset{121}{\underset{i=1}{\sum }}w_{i}{}^{2}n_{\lambda_{i}}{}^{2}\right] \end{array}$$

Therefore, the S∕N of the $y_{MRC}$ can be defined as $E\left[{\left(\overset{121}{\underset{i=1}{\sum }}w_{i}s_{\lambda_{i}}\right)}^{2}\right]/E\left[\overset{121}{\underset{i=1}{\sum }}w_{i}{}^{2}n_{\lambda_{i}}{}^{2}\right]$. According to the Cauchy–Schwarz inequality and the MRC algorithm 21, the result of the $S∕N_{y_{MRC}}$ and the optimal weights can be expressed as

(5) $$\{\begin{array}{c} S/N_{y_{MRC}}\leq \overset{121}{\underset{i=1}{\sum }}S/N_{\lambda_{i}}\\ w_{i}=k\frac{E\left[s_{\lambda_{i}}\right]}{E{\left[n_{\lambda_{i}}\right]}^{2}} \end{array}$$

**Figure 9:**
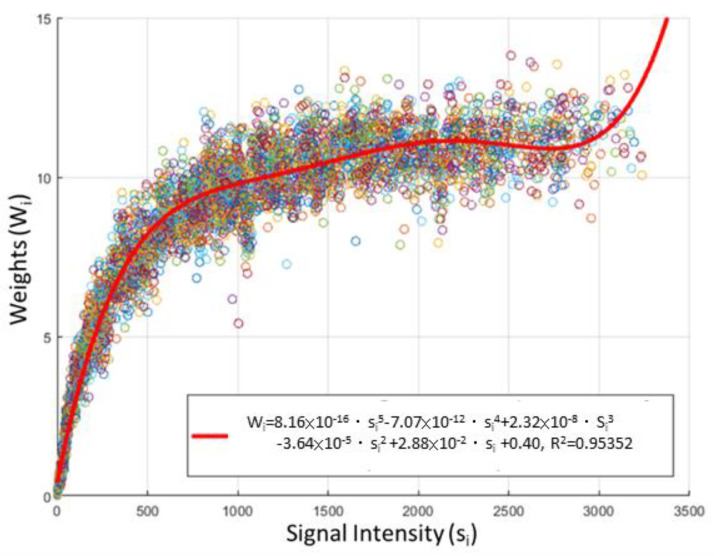
The relationship between signal intensity ($s_{i}$) and weights ($w_{i}$): the red curve represents the proposed and implemented method for the MRC weights where $w_{i}=8.16\times 10^{-16}\cdot s_{i}^{5}-7.07\times 10^{-12}\;\cdot s_{i}^{4}+2.32\times 10^{-8}\cdot s_{i}^{3}-3.64\times 10^{-5}\cdot s_{i}^{2}+2.88\times 10^{-2}\cdot s_{i}+0.40$.

As shown in Figure 9, we measured the relationship between the signal intensity and the weights through experiments and then used a fifth-order polynomial to fit an equation, where $w_{i}$ denotes the weight of *i*-th filter, and $s_{i}$ denotes the signal intensity of *i*-th filter. This proposed method for calculating weights was easy to implement and embed in the MCU for real-time measurement. Moreover, in the literature [21], it states that there is a proportional relationship between the non-pulsatile component (e.g., DC) and the pulsatile component (e.g., AC). In other words, the signal intensity could reflect the swing amplitude of the PPG signal; the larger the signal intensity, the larger the swing amplitude. Hence, the implemented weights not only achieved the optimal S/N in terms of signal intensity to noise ratio but could also be seen achieving the optimal signal to noise ratio in terms of the swing amplitude of PPG signals to the noise ratio.

Lastly, to avoid the MRC signal being amplified by the sum of weights, the normalized weights can be expressed as

(6) $${\hat{w}}_{i}=\frac{w_{i}}{\left\|w_{i}\right\|{}_{1}}=\frac{w_{i}}{\sum_{j=1}^{121}|w_{j}|}$$

A band-pass filter was used to reduce the noise of the PPG signals. The band-pass filter was designed based on the following considerations, including phase, band-pass bandwidth, attenuation of the band-pass response, and attenuation of the low-frequency response. To avoid the phase shift, we used a finite impulse response (FIR) filter. We set the passband from 0.9 to 6 Hz and cut off frequency at 7 Hz to reserve the PPG signals. To minimize the ripple effect in the passband, the ripples were limited to 0.2 dB. In order to filter out the low frequencies, we set a lower cutoff frequency at 0.1708 Hz such that the zero points could be closer to 0 Hz.

## 4. Experiment Results

**Figure 10:**
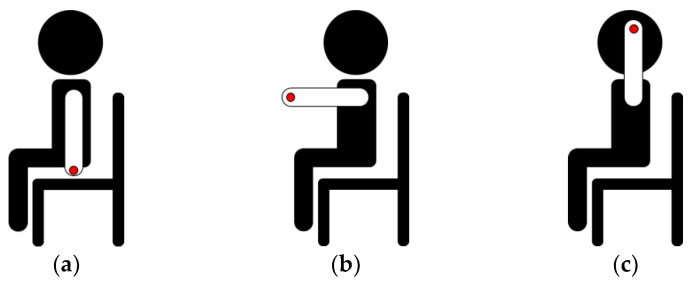
The three different postures for examining PPG measurement at different hydrostatic pressures: (**a**) Posture 1—hands down (−90°); (**b**) Posture 2—hands forward (0°); (**c**) Posture 3—hands up (+90°).

We used the developed AW-PPG device and AFE4404EVM (Texas Instruments) to record the PPG signals in three different postures to examine different hydrostatic pressures, as shown in Figure 10. Posture 1 was to put the hand below the heart. Posture 2 was to put the hand at the same height as the heart Posture 3 was to put the hand above the heart. The PPG signal was recorded for 1 min from the finger at each posture. The subject was allowed to rest for 1 min between two postures. A band-pass filter with a passband of 0.9 to 6 Hz was used to reduce the out-of-band noise.

**Figure 11:**
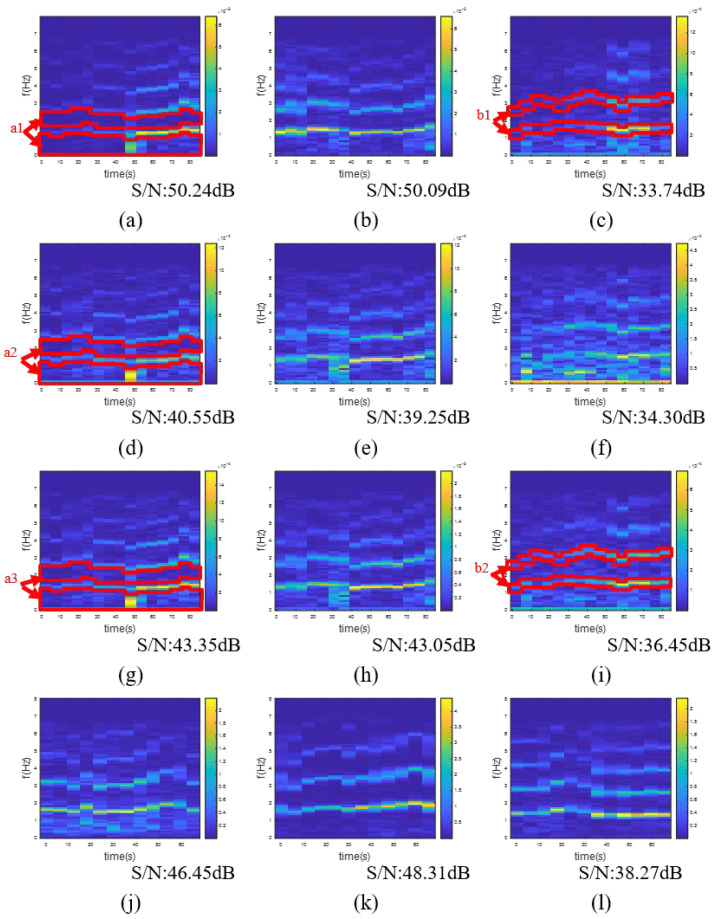
Short-time Fourier transform (STFT) of PPG signals in different postures and devices: (**a**) Green PPG of AFE4404EVM in hands down posture; (**b**) Green PPG of AFE4404EVM in hands forward posture; (**c**) Green PPG of AFE4404EVM in hands up posture; (**d**) Red PPG of AFE4404EVM in hands down posture; (**e**) Red PPG of AFE4404EVM in hands forward posture; (**f**) Red PPG of AFE4404EVM in hands up posture; (**g**) NIR PPG of AFE4404EVM in hands down posture; (**h**) NIR PPG of AFE4404EVM in hands forward posture; (**i**) NIR PPG of AFE4404EVM in hands up posture; (**j**) The proposed AW-PPG device in hands down posture; (**k**) The proposed AW-PPG device in hands forward posture; (**l**) The proposed AW-PPG device in hands up posture.

We used the developed device to record the MRC-AW-PPG signals, and then used AFE4404EVM as a reference to record the green, red, and NIR PPG signals separately, all in the three different postures. Short-time Fourier transform (STFT) was used to inspect signal qualities. We set the window size to be 10 s (909 points) and the step size to be 5 s (454 points), as shown in Figure 11. In the hands down posture, the green PPG signal showed a higher *S*/*N* of 50.24 dB than the red PPG signal of 40.55 dB and the NIR PPG signal of 43.35 dB. The higher *S*/*N* as explained by the STFT graph as the “a2” and “a3” regions showed more noise than the “a1” region. By contrast, in the hands up posture, the green PPG signal showed a lower *S*/*N* of 33.74 dB than the NIR PPG signal of 36.45 dB. The lower *S*/*N* was explained by the STFT graph as the “b1” region had a weaker fundamental frequency component and harmonic frequency component than that of the “b2” region. It was clearly observed that each posture had its most suitable wavelength. Hence, in this work, MRC-AW-PPG was proposed to be used to try to overcome different measurement conditions by supporting all-wavelength measurement. As shown in the bottom row in Figure 11, the developed MRC-AW-PPG delivered a decent *S*/*N* in the hands down, hands forward, and hands up postures with 46.5 dB, 48.3 dB, and 38.2 dB, respectively. In addition to this, a clear fundamental frequency component and harmonic frequency component were observed.

As shown in Table 1, six subjects were tested and the signal-to-noise ratio ($S/N{)}_{PPG}=\;\frac{S_{PPG}}{N_{PPG}}$ was computed, where

(7) $$\{\begin{array}{c} S_{PPG}=\int_{f>0}^{f\leq 7}Y_{PPG}{\left(f\right)}^{2}\\ N_{PPG}=\int_{f>7}Y_{PPG}{\left(f\right)}^{2}\\ Y_{PPG}\left(f\right)\;\mathrm{denotes}\;\mathrm{the}\;\mathrm{frequency}\;\mathrm{domain}\;\mathrm{amplitude},\;\\ \mathrm{derived}\;\mathrm{from}\;\mathrm{Fourier}\;\mathrm{transform}\;\mathrm{of}\;\mathrm{the}\;\mathrm{PPG}\;\mathrm{signals} \end{array}$$

As we expected, the case of hands forward had the highest ${\left(S/N\right)}_{PPG}$.

**Figure 12:**
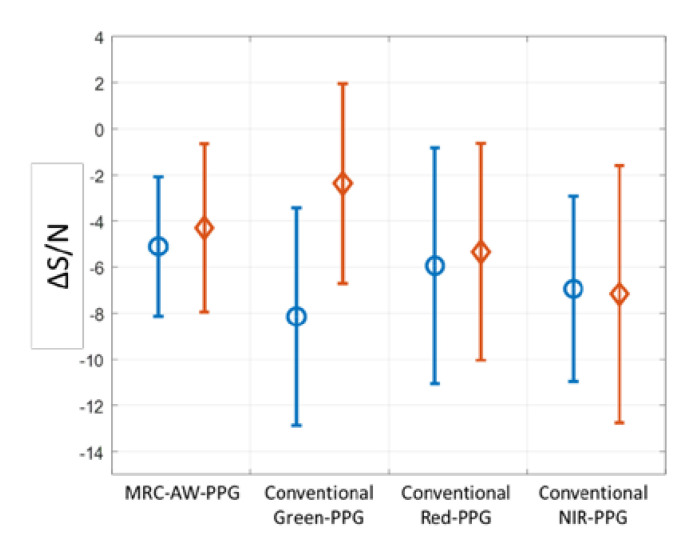
The ∆S/N of the proposed MRC-AW-PPG against conventional green, red, and near-infrared (NIR) PPG sensors. 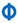 means difference between hands forward and hands up; 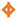 means difference between hands forward and hands down.

Due to the variety and complexity of skin tissue and the difficulty of maintaining consistent experimental measurements in a few cases, for example, subject 3, we observed that the performance of the proposed AW-PPG was worse than that of the TI’s device. However, it was observed that the overall performance was better in an average sense. The preliminary experimental results show that the ∆S/N (the difference between hands forward with hands down and hands up) of MRC-AW-PPG and conventional green, red, and NIR PPG signals are −4.30 ± 3.99/−5.10 ± 3.31 dB, −2.37 ± 4.74/−8.14 ± 5.17 dB, −5.34 ± 5.15/−5.94 ± 5.58 dB and −7.17 ± 6.11/−6.94 ± 4.40 dB, respectively, as shown in Figure 12. The MRC-AW-PPG signal gave higher adaptability in different hydrostatic pressure conditions. This inferred that although the best depth to get the clearest signal was unknown when the hydrostatic pressure was present, the AW-PPG device could record the signal of various depths and then use the MRC algorithm to combine the pulsatile signal of each wavelength, resulting in stable signals.

Assuming the signal of hands forward to be the standard signal, we noted that the standard signal coming from the proposed AW-PPG device was a bit worse than that coming from TI’s device. This could have been due to the hardware specifications of these two devices. The quantization level of the implemented AW-PPG device is 12-bits mapping to 0 to 3.3 V, so that its quantization error is $3.3/2^{12}.$ The quantization level of TI is 22-bits mapping to 0 to 1.2 V, so that its quantization error is $1.2/2^{22}$. We expect that the performance of the implemented device can be greatly improved when the quantization level is increased.

## 5. Conclusions

In this work, we developed an all-wavelength photoplethysmography (AW-PPG) device and integrated it with a wireless Bluetooth Low Energy (BLE) transmission and a mobile app to form a convenient portable AW-PPG measurement system. We exploited the computing power of the mobile device to acquire the PPG signal, from 400 nm to 1000 nm synchronously, in real time, and calculated the physiological information. By probing pulsatile signals from various depths of subcutaneous tissue, we observed potentials of AW-PPG measurement. The PPG signals from the developed AW-PPG device versus those from the conventional SW-PPG device were compared in terms of hydrostatic pressure conditions. It has been observed that the developed AW-PPG device can provide more stable PPG signals than that of a conventional PPG device. The results shine a light on the potential benefits of using multiple wavelengths for the next generation of noninvasive PPG sensing. A larger scale Institutional Review Boards (IRB) approved test is planned.

## Figures and Tables

**Table 1:** ${\left(S/N\right)}_{PPG}$ of six subjects in the three measurement conditions.

	Device	Light	A. Hands Up	B. Hands Forward	C. Hands Down	Δ*S*/*N* A-B (dB)	Δ*S*/*N* C-B (dB)
Subject 1	Proposed AW-PPG	AW	39.05	41.72	42.00	−2.67	0.28
	AFE4404EVM	Green	40.88	49.46	47.42	−8.57	−2.03
		Red	42.16	47.17	43.65	−5.01	−3.52
		IR	41.00	47.59	44.25	−6.60	−3.34
Subject 2	Proposed AW-PPG	AW	42.00	46.40	40.65	−4.40	−5.76
	AFE4404EVM	Green	30.05	35.76	40.48	−5.71	4.72
		Red	25.44	40.15	35.56	−14.72	−4.59
		IR	28.40	42.76	38.79	−14.36	−3.97
Subject 3	Proposed AW-PPG	AW	43.90	48.49	38.51	−4.59	−9.98
	AFE4404EVM	Green	46.94	47.44	43.83	−0.50	−3.61
		Red	46.46	47.51	41.81	−1.04	−5.69
		IR	47.13	47.62	39.36	−0.49	−8.26
Subject 4	Proposed AW-PPG	AW	38.27	48.31	46.45	−10.04	−1.86
	AFE4404EVM	Green	33.74	50.09	50.24	−16.35	0.14
		Red	34.30	39.25	40.55	−4.95	1.30
		IR	36.45	43.05	43.45	−6.60	0.39
Subject 5	Proposed AW-PPG	AW	39.43	47.27	40.00	−7.84	−7.27
	AFE4404EVM	Green	42.85	51.05	47.16	−8.20	−3.89
		Red	41.89	51.93	37.41	−10.04	−14.52
		IR	46.29	52.95	36.53	−6.66	−16.41
Subject 6	Proposed AW-PPG	AW	44.85	45.91	44.69	−1.06	−1.23
	AFE4404EVM	Green	46.14	55.65	46.08	−9.51	−9.57
		Red	45.99	45.89	40.86	0.10	−5.03
		IR	46.36	53.29	41.86	−6.94	−11.43
